# Determinants of Sleep Quality in Inflammatory Bowel Diseases

**DOI:** 10.3390/jcm9092921

**Published:** 2020-09-10

**Authors:** Marcin Sochal, Ewa Małecka-Panas, Agata Gabryelska, Renata Talar-Wojnarowska, Bartosz Szmyd, Monika Krzywdzińska, Piotr Białasiewicz

**Affiliations:** 1 Department of Sleep Medicine and Metabolic Disorders, Medical University of Lodz, 92-215 Lodz, Poland; agata.gabryelska@gmail.com (A.G.); bartoszmyd@gmail.com (B.S.); piotr.bialasiewicz@umed.lodz.pl (P.B.); 2 Department of Digestive Tract Diseases, Medical University of Lodz, 90-153 Lodz, Poland; ewuncia@poczta.onet.pl (E.M.-P.); r-wojnarowska@wp.pl (R.T.-W.); monika.krzywdzinska@wp.pl (M.K.)

**Keywords:** inflammatory bowel diseases, sleep disorders, sleep medicine

## Abstract

The causes of disordered sleep, frequently reported by patients with inflammatory bowel diseases (IBD), are poorly understood. The study aimed to evaluate sleep quality in IBD patients and to identify factors affecting their sleep. IBD patients (n = 133) and healthy controls (HC; n = 57) were included in the study and completed sleep questionnaires (Pittsburgh Sleep Quality Index (PSQI), Athens insomnia scale (AIS), and Epworth sleepiness scale (ESS)), Beck Depression Inventory (BDI), and pain scales (Visual Analogue Scale and Laitinen Pain Scale). IBD patients attained higher scores in all sleep questionnaires compared to HC: PSQI, AIS, and ESS (all *p* < 0.001). They also had prolonged sleep latency (*p* < 0.001) with reduced sleep efficiency (*p* < 0.001). Patients in exacerbation of IBD had higher scores in PSQI (*p* = 0.008), ESS (*p* = 0.009), but not in AIS, compared to those in remission. Participants with comorbid chronic diseases had higher scores in PSQI and AIS, but not in ESS, compared to others. Multiple regression revealed that the sleep questionnaire results were significantly affected by mood level (BDI), but not by the aforementioned pain scales. Sleep impairment in IBD patients is a common problem that deserves attention in everyday clinical practice and mood level seems to be the main factor affecting the quality of sleep in IBD patients.

## 1. Introduction

Inflammatory bowel diseases (IBD) represent the group of chronic gastrointestinal tract diseases, including Crohn’s disease (CD) and ulcerative colitis (UC) [1,2,3]. Impaired regulation of local and systemic immunologic reactions, as well as changes in the intestinal microbiome, have been implicated in their etiology [4]. Due to the high prevalence of IBD in developed countries, it is proposed that such factors as stress, obesity, lack of exercise, anxiety, or sleep disorders may be important in the IBD clinical course and etiopathogenesis [5].

Sleep is a physiological state in which people spend over one-third of their life, but so far it is poorly understood [6,7]. The association between IBD and sleep disturbances is bidirectional, i.e., disease exacerbation leads to sleep disturbances, and the latter increases the disease activity [8]. Sleep may interfere with the gut-brain axis. This axis can be affected by changes in cortisol levels that reach a nadir at the beginning of sleep and a peak before awakening [9]. Moreover, during sleep, the activity of the complement system is increased, and immunological memory is formed [10]. In the dextran sodium sulfate-induced mouse model of UC, the increase in colonic inflammation has been observed following intermittent sleep deprivation [11]. It has been proven that partial sleep deprivation affects the immune system, increasing susceptibility to infection and reducing the immune response to vaccinations [12]. Conversely, inflammation can affect the quality of sleep. For instance, administration of interleukin-1-β (IL-1-β), tumor necrosis factor (TNF), or interferon-α (IFN-α) into the cerebral ventricle of rabbits enhances the non-rapid eye movement sleep (NREMS) phase [13].

Numerous studies on sleep quality among IBD patients have been published [14,15,16]. There have been, however, limited reports comparing disordered sleep in IBD and healthy control (HC). Insomnia symptoms among IBD patients have not been investigated by a dedicated scale yet. Most researchers used only one scale to assess disordered sleep, e.g., Pittsburgh Sleep Quality Index (PSQI), which is a validated and widely used questionnaire; however, the overall score does not indicate the plausible causes of sleep disturbances, such as difficulty in falling asleep, waking up at night, or reduction of sleep efficiency [17]. There have been only a few reports on the origin of disordered sleep among IBD patients, which may contribute to the more effective treatment thereof in this group of patients [15,18].

Therefore, the aims of the study were assessment of sleep quality and the search for factors affecting sleep among IBD patients.

## 2. Experimental Section

### 2.1. Sample

There were 190 study participants recruited for the study in the Department of Digestive Tract Diseases, Medical University of Lodz, Poland, which included 133 IBD patients (68 with CD and 65 with UC) and 57 apparently healthy subjects serving as control (HC).

The severity of the disease was assessed by clinical scales: Harvey–Bradshaw Index (HBI) for CD and Partial Mayo Score (PMS) for UC. Disease remission was defined as a score below 5 points according to HBI and 2 points according to PMS [19,20].

Information on the course of the disease, previous gastrointestinal operations, the presence of fistulas (perianal, enteroureteral, enterovaginal, and/or enteroenteric), perianal fissures, abscesses, the disease onset, extraintestinal complications (arthritis, iritis and scleritis, acute pancreatitis, hepatitis or erythema nodosum), information about the current treatment, smoking and other chronic diseases (such as asthma, compensated hypothyroidism, migraine, musculoskeletal system diseases, diabetes, endometriosis, rheumatoid arthritis, and psoriasis) were collected.

The inclusion criteria comprised of the signing of informed consent to participate in the study, age over 18 and under 65, and diagnosis of IBD based on clinical, radiological, endoscopic, and histopathological criteria. Exclusion criteria were previous abdominal and thoracic surgery in the last six months, active malignant disease except for skin basal cell carcinoma, addiction to alcohol or other psychoactive substances, diagnosed and treated psychiatric disorders.

Healthy volunteers were recruited for the study according to the snowball sampling method [21]. The additional criteria for inclusion in the healthy group included: no history of chronic diseases (apart from hypertension or hypercholesterolemia), especially those related to the digestive system, and no history of hypnotic drugs use. Health control was matched to IBD patients in terms of sex, age, and BMI.

The study protocol was accepted by the Ethical Committee of the Medical University of Lodz, Poland (number: RNN/433/18/KE). All respondents received information for the patient and were asked to sign informed consent to participate in the study.

### 2.2. Questionnaires

Respondents completed questionnaires of sleep quality, mood level, and pain intensity. The study was conducted in a ward and outpatient clinic, which are part of the Department of Digestive Tract Diseases. The researchers provided participants with instructions regarding the questionnaires. All patients who consented to participate in the study, completed all questionnaires. Sleep quality was measured by PSQI [17], Athens Insomnia Scale (AIS) [22,23], and Epworth sleepiness scale (ESS) [24]. Obtaining 6 or more points in PSQI was considered as reduced sleep quality, >5 in AIS as mild insomnia, and >10 points in ESS as drowsiness. Sleep efficiency was expressed as a total sleep time related to time spent in bed. To assess the subjective severity of pain, Visual Analogue Scale (VAS), and Laitinen Pain Scale (LPS) were used [25,26]. The mood level was measured by the Beck Depression Inventory (BDI). 

### 2.3. Statistical Analysis

Statistical analysis was performed using Statistica 13.1PL (StatSoft, Tulsa, OK, USA). Obtained data had non-normal distribution (Shapiro–Wilk test, *p* < 0.05). Thus, they were presented as median with interquartile range (IQR: first–third quartile) and non-parametric tests were used: Mann–Whitney U test for two independent samples, and Spearman’s rank correlation for two continuous variables. Fisher’s exact test (n_min_ < 5), χ2 with Yates’ correction (5 ≤ n_min_ < 15), and χ2 (n_min_ ≥ 15) were used for testing dependencies between nominal data. Moreover, we decided to conduct a multiple regression analysis (a forward stepwise model) to determine the impact of dependent data, including BDI and pain parameters (LPS, VAS), on selected sleep questionnaires (PSQI, AIS, and ESS). A *p*-value less than 0.05 was considered as statistically significant; only for multiple testing between dependent subgroups the Bonferroni correction was used (*p*-value divided by the number of tests between subgroups).

## 3. Results

Eighty-three patients (62.4%) suffered from active IBD, while 50 participants (37.6%) were in remission. The characteristics of the study participants are presented in Table 1.

IBD patients attained higher scores in all sleep questionnaires compared to HC. They also had prolonged sleep latency, more often complained of nocturnal defecation, and their sleep efficiency was reduced compared to HC (Table 2). Six or more points in the PSQI scale were obtained by 43.6% of IBD patients (n = 58) vs. 26.3% of HC (n = 15, *p* = 0.025). Similarly, higher percentage of IBD patients scored more than 5 points in AIS, namely 49.6% (n = 66) vs. 26.3% of HC (n = 15, *p* = 0.003). Further, at least 11 points in ESS was attained by 21.1% of IBD patients (n = 28) while only by 5.3% of HC (n = 4, *p* = 0.004).

Results of sleep questionnaires were similar in CD and UC patients: PSQI (5, 4–8 vs. 5, 4–7, *p* = 0.740), AIS (6, 4–8 vs. 5, 3–8, *p* = 0.335), and ESS (6, 4–8.25 vs. 6, 4–9, *p* = 0.692), respectively.

Patients in exacerbation of IBD had higher scores in PSQI and ESS, but not in the AIS, compared to the remission. Sleep latency and efficiency were similar in both groups, but patients in exacerbation were more often presented with nocturnal defecation, Table 2.

Patients in exacerbation of IBD had higher scores in sleep questionnaires, longer sleep latency time, lower sleep efficiency, and more often waking up in the night than HC, Table 2.

Patients in IBD remission had similar sleep questionnaire scores and the frequency of waking up in the night, but have longer sleep latency and their sleep efficiency was lower compared to HC, Table 2.

HBI values (estimated for CD patients) were positively correlated with PSQI (r = 0.390, *p* = 0.001), but not with AIS (r = 0.163, *p* = 0.183), nor ESS (r = 0.183, *p* = 0.135). There were also positive correlations between PMS (estimated for UC patients) and PSQI (r = 0.256, *p* = 0.040), AIS (r = 0.370, *p* = 0.002), as well as ESS (r = 0.276, *p* = 0.026). The age of IBD patients did not correlate with the PSQI (r = 0.091, *p* = 0.288), AIS (r = 0.042, *p* = 0.629), and ESS (r = −0.049, *p* = 0.569) scores.

The following factors that may have affected the results of sleep questionnaires were identified: Patients with comorbid chronic diseases and taking hypnotic drugs had higher scores in PSQI and AIS, but not in ESS, compared to others. Steroid therapy did not affect the results of PSQI, AIS, and ESS. There was no difference in the results of PSQI, AIS, and ESS among patients with previous abdominal surgery, perianal fistulas, and treated with immunomodulatory drugs (Table 3).

Positive correlations have been shown between scores of sleep scales (PSQI, AIS, and ESS) and the severity of depression according to BDI as well as pain according to VAS and LPS, Table 4. Multiple regression, using the progressive step method, revealed that sleep questionnaire results were significantly affected by BDI mood level, but not by the aforementioned pain scales scores. Obtained models explain 49.0% variability of PSQI, 39.1% of AIS, and 9.2% of ESS (Table 4).

## 4. Discussion

The problem of disordered sleep in immune diseases has been recently widely studied [14,16,27,28]. To date, only a few studies have been published assessing sleep quality among IBD patients using mainly PSQI [14,29]. The impact of factors such as pain and depression on sleep in patients with IBD has not been extensively evaluated yet.

In our study, patients with CD and UC had similar sleep quality measured with PSQI and AIS, and daytime sleepiness according to ESS. Sobolewska et al. also did not notice differences in the quality of sleep between CD and UC on a small group of patients measured by PSQI [14]. Interestingly, Ananthakrishnan et al. showed, that reduced quality of sleep in CD patients was a risk factor of exacerbation within 6 months, in contrast to UC patients, in whom no such association was demonstrated. CD patients had a slightly higher risk of developing disordered sleep compared to UC [15].

We showed, that IBD patients had greater sleep-related problems measured by PSQI compared to HC. The severity of the disease had an impact on the results of PSQI and patients in exacerbation had worse sleep quality compared to those in remission and HC. In another study, the decreased quality of sleep measured by PSQI in CD patients compared to healthy controls was also noted and the correlation between the severity of the disease and the PSQI score was observed [18]. We did not notice the difference in the sleep duration either between patients with IBD and HC or between patients in disease exacerbation and remission. On the other hand, Gingold-Belfer et al. observed differences in the length of sleep duration between patients in exacerbation, remission, and HC. In contrast to our study, there was no difference in sleep latency and sleep efficiency [18]. We observed that sleep efficiency was worse in IBD than HC, but did not differ between the exacerbation and remission subgroups. It should be noted that the results obtained can be significantly biased by the subjective nature of the data. Paixão et al. showed in a small group of IBD patients (n = 20) differences in sleep efficiency between patients in exacerbation and remission assessed by PSQI, but not in polysomnography examination [30]. Polysomnography is not dedicated to the diagnosis of all sleep disorders, i.e., sleep diary, questionnaires, and actigraphy could be more helpful in the diagnosis of insomnia [31]. However, polysomnography examination on a large group of IBD could provide additional information about the nature of the sleep disturbances in this group of patients.

Additionally, we noticed more frequent sleep interruption by getting up to the toilet in IBD patients compared to HC, as well as among patients in IBD exacerbation than in remission. Therefore, unsurprisingly, diarrhea may be one of the main causes of disordered sleep.

Although many guides for patients highlights the problem of insomnia in IBD, the studies using scales dedicated to its evaluation are few. In the Canadian Community Health Survey, the likelihood of bowel disorders increased with the frequency of insomnia problems; however, the severity of insomnia was measured using questions elaborated by the authors [32]. Recently, we noticed a positive correlation between brain-derived neurotrophic factor serum level and the results of the AIS questionnaires among CD patients [33]. In another study in young patients, no differences in the severity of insomnia were observed in relation to the clinical state of IBD [34]. However, to assess insomnia, the Women’s Health Initiative Insomnia Rating Scale was used, which was validated only in postmenopausal women [35]. In our study, the symptoms of insomnia (according to the AIS) were present more often among IBD patients than in HC. AIS results correlated with the clinical severity of CD, but not UC. Some authors postulate a two-way interaction between sleep quality and disease severity [32,36]. The colitis mouse model suggests that insomnia may exacerbate the disease. Acute sleep deprivation increased disease severity measured with tissue myeloperoxidase and chronic intermittent sleep deprivation caused both worsening of histological as well as clinical manifestations of colitis [11]. A study of a cohort group including 151,871 women shown that the duration of sleep between 6 and 9 h per day reduced risk for the development of UC, but interestingly, not for CD [16]. Shorter sleep time is associated with higher TNF level, while longer sleep time with an increased level of C-reactive protein and IL-6 [8,37]. Sleep deprivation causes many immunological phenomena that can affect the course of IBD; for example, TNF is secreted during sleep deprivation, which is a target for effective anti-TNF therapy of IBD [38,39,40].

Patients in our study attained higher median results of ESS reflecting daytime sleepiness than HC. However, only 21% of IBD patients scored 11 or more points (which characterizes at least mild sleepiness). Iskandar et al. did not notice the difference in ESS between CD and HC, but patients with active disease had higher scores than in the remission. There were also no differences in the results of actigraphy and urine melatonin levels between CD and controls [27]. In another study, there were no differences in ESS depending on the severity of the disease, while actigraphy has shown decreased sleep efficiency in moderate and severe CD group compared to patients in remission [34]. It is worth mentioning that urine melatonin levels and actigraphy are not dedicated methods to assess sleepiness. Clinical scales are used to screen for hypersomnia, but the gold standard is the Multiple Sleep Latency Test [41]. Perhaps ESS is not a fully reliable tool for assessing sleepiness in IBD patients. This scale was created to assess drowsiness in the course of sleep disorders, such as narcolepsy, other hypersomnias of the central origin, or obstructive sleep apnea [24,42]. On the other hand, ESS is simple and easy to use in everyday clinical practice.

Sleep is affected by many factors, such as mood, stress, and medications. One of them is the use of glucocorticosteroids, their negative effects on sleep have been reported [43]. Nevertheless, we found that patients on steroid therapy had similar sleep quality according to PSQI, AIS, and ESS. On the other hand, Ananthakrishnan et al. noticed the negative effect of steroids on the quality of sleep in IBD patients [15]. Additionally, we found that IBD patients with coexisting chronic diseases had decreased quality of sleep, which is expected as multiple comorbidities are a known risk factor for reduced sleep quality [44]. Surprisingly, the presence of fistulas and history of abdominal surgery did not affect the results obtained in the sleep quality questionnaires.

Disordered sleep may also be affected by the concurrent depression, which in the patients with IBD is more often diagnosed than in the general population [45,46,47]. Numerous studies have been published assessing the frequency of depression in IBD patients [46,47]. This disorder had been often diagnosed one year before IBD diagnosis [46]. In our study, patients with a history of mental illness were excluded from the analysis, but many of them might have undiagnosed disorders, especially depression. We noticed a strong correlation between the level of depression measured with the BDI questionnaire and the scores of other sleep scales—PSQI, AIS, and ESS. It has been previously shown that patients with depressive symptoms had an almost three-fold increased risk of sleep disturbances, which is consistent with the results of our study [15]. 

Pain also represents an important factor affecting sleep quality in the general population [48]. We have also shown that the subjective severity of pain was associated with higher sleep questionnaire scores. The resulting multiple regression model explained almost 50% of the PSQI variability with the mood level as a significant variable, while, quite surprisingly, the reported severity of pain did not affect sleep quality. 

The limitation of this study was the use of subjective methods to assess the quality of sleep. On the other hand, the questionnaires used were validated and are still widely used. The use of three questionnaires assessing sleep in its various aspects increased the value of information obtained. However, future research should also include polysomnography and actigraphy to better understand the nature of sleep disturbances in IBD patients. The division of patients by clinical condition based on subjective scales (HBI and PMS) was also a limitation to this study. However, these scales are based on the complaints reported by patients that have an important impact on their perception of the quality of life.

## 5. Conclusions

Sleep impairment in IBD patients is a common problem that deserves attention in everyday clinical practice. Adequate sleep counseling can not only improve the quality of life of these patients, but also can have a positive effect on the course of the disease. Mood level, but not pain, is the main factor affecting the quality of sleep in IBD patients. Future research should focus on the search for causes of reduced quality of sleep and their impact on the risk of morbidity or exacerbation of the disease.

## Figures and Tables

**Table 1:** Characteristics of the participants.

Parameter	IBD			HC	*p*	
	All	Exacerbation	Remission			
n (%)	133 (70%)	83 (44%)	50 (26%)	57 (30%)	-	
CD	68 (51%)	40 (59%)	28 (41%)	-	-	
UC	65 (49%)	42 (65%)	23 (35%)			
HBI	5 (2–8)	7 (6–9)	2 (1–3)	-	-	
PMS	3 (1–4)	4 (3–5.75)	1 (0–1)			
n women (%)	73 (55%)	40 (48%)	33 (66%)	33 (58%)	^1^ 0.702 ^3^ 0.259	^2^ 0.422 ^4^ 0.752
Age	37.0 (30.0–47.0)	36.0 (30.0–46.5)	38.0 (28.5–46.8)	38.0 (28.0–50.0)	^1^ 0.899 ^3^ 0.882	^2^ 0.754 ^4^ 0.958
BMI (kg/m^2^)	23.5 (20.5–26.5)	23.5 (20.8–27.4)	23.2 (20.3–26.1)	24.2 (21.8–25.7)	^1^ 0.394 ^3^ 0.546	^2^ 0.736 ^4^ 0.341
Hypnotic drugs	18 (13.5%)	15 (18.1%)	3 (6.0%)	-		^2^ 0.066
Steroids treatment	33 (25%)	33 (40%)	0 (0%)	-	-	
Immuno-modulators	45 (34%)	28 (34%)	17 (34%)	-	-	
Anti-TNF therpay	29 (22%)	29 (35%)	0 (0%)	-	-	

Data are presented as median (IQR) unless otherwise indicated. In bold—statistically significant differences regarding multiple testing correction between subgroups; ^1^ All vs HC; ^2^ Exacerbation vs Remission; ^3^ Exacerbation vs HC; ^4^ Remission vs HC; Abbreviations: BMI: body mass index; CD: Crohn’s disease; ESS: Epworth sleepiness scale; HBI: Harvey–Bradshaw Index; HC: health control; IBD: inflammatory bowel diseases; IQR: interquartile range; n: number; PMS: Partial Mayo Score; TNF: tumour necrosis factor; UC: ulcerative colitis.

**Table 2:** Comparison of the results of questionnaires/scales between study groups.

Parameter	IBD			HC	*p*	
	All	Exacerbation	Remission			
PSQI	5 (4–7)	5 (4–8)	5 (3–6.8)	4 (2–6)	^1^ **<0.001** ^3^ **<0.001**	^2^**0.008** ^4^ 0.067
ESS	6 (4–9)	6 (4–10.5)	5 (3–7)	3 (2–7)	^1^ **<0.001** ^3^ **<0.001**	^2^**0.009** ^4^ 0.105
AIS	5.0 (4.0–8.0)	6.0 (4.0–9.0)	5.0 (3.0–7.0)	3.0 (1.0–6.0)	^1^ **<0.001** ^3^ **<0.001**	^2^ 0.021 ^4^ 0.019
BDI	7.0 (4.0–11.0)	8.0 (5.0–12.0)	5.0 (2.0–9.8)	2.0 (1.0–4.0)	^1^ **<0.001** ^3^ **<0.001**	^2^ **0.001** ^4^ **0.001**
VAS	4.0 (0–5.0)	5.0 (2.0–5.5)	2.8 (0–4.9)	0 (0–3.0)	^1^ **<0.001** ^3^ **<0.001**	^2^ **0.004** ^4^ **0.009**
LPS	3.0 (0.0–5.0)	4.0 (2.0–6.0)	2.0 (0.0–3.0)	1.0 (0.0–3.0)	^1^ **0.004** ^3^ **<0.001**	^2^**0.001** ^4^ 0.617
Sleep latency [min]	15 (10–30)	15 (10–35)	15 (10–30)	10 (5–15)	^1^ **<0.001** ^3^ **<0.001**	^2^ 0.352 ^4^ **0.009**
Hours of sleep	7.0 (6.0–7.8)	7.0 (6.0–8.0)	7.0 (6.0–7.5)	6.8(6.0–7.3)	^1^ 0.304 ^3^ 0.381	^2^ 0.968 ^4^ 0.352
Hours spent in bed	8.0 (7.0–8.5)	8.0 (7.0–8.5)	8.0 (7.0–8.5)	7.0 (6.5–8.0)	^1^ **<0.001** ^3^ **<0.001**	^2^ 0.824 ^4^ **0.002**
Sleep efficiency (%)	88.9 (83.2–94.7)	88.9 (81.8–95.5)	89.4 (86.2–94.4)	97.2 (92.9–98.6)	^1^ **<0.001** ^3^ **<0.001**	^2^ 0.662 ^4^ **<0.001**
Waking up in the night n (%)	94 (70.7%)	66 (79.5%)	28 (56.0%)	24 (42.1%)	^1^ **<0.001** ^3^ **<0.001**	^2^**0.004** ^4^ 0.151

Data are presented as median (IQR) unless otherwise indicated. In bold—statistically significant differences regarding multiple testing correction between subgroups; ^1^ All vs HC; ^2^ Exacerbation vs Remission; ^3^ Exacerbation vs HC; ^4^ Remission vs HC; Abbreviations: AIS: Athens Insomnia Scale; BDI: Beck Depression Inventory; ESS: Epworth sleepiness scale; HC: health control; IBD: inflammatory bowel diseases; IQR: interquartile range; LPS: Laitinen Pain Scale; n: number; PSQI: Pittsburgh Sleep Quality Index; VAS: Visual Analogue Scale.

**Table 3:** Factors affecting the quality of sleep.

Factor		PSQI		AIS		ESS	
Steroids treatment	Yes	5.0 (4.0–9.0)	*p* = 0.210	6.0 (4.0–9.0)	*p* = 0.410	6.0 (4.0–8.0)	*p* = 0.395
	No	5.0 (4.0–7.0)		5.0 (3.0–8.0)		6.0(3.0–9.0)	
Hypnotic drugs	Yes No	10.0 (7.0–15.0) 5.0 (4.0–7.0)	*p* < 0.001	7.0 (5.0–10.0) 5.0 (3.0–8.0)	*p* = 0.040	6.0 (3.0–8.0) 6.0 (4.0–9.0)	*p* = 0.808
Abdominal surgery	Yes	5.0 (4.0–8.0)	*p* = 0.501	7.0 (4.0–9.0)	*p* = 0.306	5.0 (4.0–8.0)	*p* = 0.424
	No	5.0 (4.0–7.0)		5.0 (3.3–8.0)		6.0 (4.0–9.8)	
Comorbid chronic diseases *	Yes	5.0 (4.0–7.0)	*p* = 0.044	6.0 (3.0–8.0)	*p* = 0.049	6.0 (3–8.5)	*p* = 0.191
	No	5.0 (3.0–7.0)		5.0 (2.5–7.0)		5.0 (3.0–8.0)	
Fistulas	Yes	5.0 (3.0–12.0)	*p* = 0.630	6.0 (4.0–8.5)	*p* = 0.880	4.0 (3.0–5.0)	*p* = 0.057
	No	5.0 (4.0–7.0)		5.0 (4.0–8.0)		6.0 (4.0–9.0)	
Immuno-modulators	Yes	5.0 (4.0–7.0)	*p* = 0.652	6.0 (4.0–7.0)	*p* = 0.841	6.0(4.0–11.0)	*p* = 0.532
	No	5.0 (4.0–7.25)		5.0 (3.0–9.0)		6.0 (4.0–8.0)	
Anti-TNF therapy	Yes	5.0 (4.0–8.0)	*p* = 0.863	5.0 (4.0–8.0)	*p* = 0.575	6.0(4.0–9.0)	*p* = 0.833
	No	5.0 (4.0–7.0)		5.5 (4.0–8.0)		6.0 (4.0–8.25)	

Data are presented as median (IQR). * such as asthma, compensated hypothyroidism, hypertension, migraine, musculoskeletal system diseases, diabetes, endometriosis, rheumatoid arthritis, and psoriasis; Abbreviations: AIS: Athens Insomnia Scale; ESS: Epworth sleepiness scale; PSQI: Pittsburgh Sleep Quality Index; TNF: tumour necrosis factor.

**Table 4:** Association between sleep questionnaires results, pain scales, and depression rate.

	Correlation		Regression	
PSQI				
BDI	r = 0.540	*p* < 0.001	b = 0.316	*p* < 0.001
VAS	r = 0.286	*p* = 0.001		
LPS	r = 0.324	*p* < 0.001		
*R^2^ = 0.490*, *b* = *3.264*, *p* < 0.001				
AIS				
BDI	r = 0.533	*p* < 0.001	b = 0.276	*p* < 0.001
VAS	r = 0.327	*p* < 0.001	b = 0.139	*p* = 0.195
LPS	r = 0.367	*p* < 0.001		
*R^2^ = 0.391*, *b* = *3.318*, *p* < 0.001				
ESS				
BDI	r = 0.296	*p* = 0.001	b = 0.129	*p* = 0.027
VAS	r = 0.239	*p* = 0.006		
LPS	r = 0.245	*p* = 0.004	b = 0.187	*p* = 0.238
*R^2^ = 0.092*, *b* = *5.182*, *p* < 0.001				

AIS: Athens Insomnia Scale; BDI: Beck Depression Inventory; ESS: Epworth sleepiness scale; LPS: Laitinen Pain Scale: Pittsburgh Sleep Quality Index; VAS: Visual Analogue Scale.
